# An Evaluation of the Fracture Resistance of Endodontically Treated Teeth Restored With Two Types of Posts (Diamond Posts and Fiber Posts): An In Vitro Study

**DOI:** 10.7759/cureus.50209

**Published:** 2023-12-09

**Authors:** Nawar Hussam Eddien, Aziz Abdullah

**Affiliations:** 1 Department of Conservative Dentistry and Endodontics, Tishreen University, Lattakia, SYR

**Keywords:** glass-fiber post, premolars, endodontically treated teeth, fracture resistance, diamond post

## Abstract

Objectives: The aim of this study is to compare the fracture resistance and the mode of failure of endodontically treated permanent mandibular premolars restored with two types of posts: diamond posts and glass-fiber posts. Materials and methods: Forty human mandibular premolars indicated for orthodontic extraction were collected for this study and were similar in shape, size, and length. The teeth were sectioned horizontally, 1 mm above the cementoenamel junction. Root canal treatment was carried out on all specimens, and obturation was done by lateral condensation. The teeth were randomly divided into two groups. The post space was prepared in the first group to receive the diamond posts and in the second group to receive the fiber posts. A dual-cure resin material was used for cementing the posts and building up the cores. Then, they were subjected to a compressive load at a cross-head speed of 1 mm per minute and at an angle of 135 degrees to the long axis of the tooth until a fracture occurred using a universal testing machine. Fracture resistance and the mode of failure were assessed. The fracture above the acrylic block level was considered favorable, and the fracture below the acrylic block level was considered unfavorable. The data were analyzed statistically. The statistical analysis of fracture resistance between the two groups was carried out with a t-test. The statistical analysis for the failure mode of the teeth was carried out with a chi-square test. Results: There was a statistically significant difference showing that the diamond posts had better fracture resistance when compared with glass-fiber posts, while there was no statistically significant difference in terms of the failure mode between the two posts. Conclusion: Within the limitations of this study, it can be concluded that diamond posts showed higher fracture resistance than glass-fiber posts. Glass-fiber posts showed more favorable failure modes but were statistically insignificant compared to diamond posts.

## Introduction

As a result of alterations in the physical status of pulpless teeth and damage to coronal tissues, root canal-treated teeth are frequently vulnerable to crown fracture [1]. When there is inadequate structure in the crown of the tooth to support the core, posts are often utilized for rehabilitating the teeth [2]. By evenly dispersing torquing forces into supporting tissues from the root, the post system preserves the tooth from intraoral loads. This spreads stress throughout the root. In addition, support the core that took the place of the destroyed tissue of the crown [3]. There are many different types of endodontic posts available, such as metallic and non-metallic, rigid and flexible, prefabricated and custom-made, and esthetic and non-esthetic [4]. Traditional metal custom posts, which were frequently utilized in older times, are highly laborious and do not match the tooth color [5]. Several tooth-colored posts, including glass-fiber posts and zirconia posts, were designed in response to rising requests for enhanced physical properties and appearance [6]. A variety of parameters impact the ability of a tooth restored with a post and core system to resist fracture. A number of parameters, such as post length, design and material, core type, and luting agent, are intimately linked to the post and core. Cuspal coverage, residual tissue of the crown, and loading circumstances are other parameters that are linked to the restored tooth [7]. The post-material will play a significant role in determining the durability of a root canal-treated tooth that has insufficient crown tissue [8]. As opposed to conventional metal posts, such as stainless steel and titanium, glass-fiber posts have elasticity approximately similar to that of dentine, so the risk of root fracture is minimized [9]. The biomechanical behavior of diamond posts has not been compared in published studies. Therefore, the aim of this study was to compare the fracture resistance and the mode of failure of endodontically treated permanent mandibular premolars restored with two types of posts: diamond posts and glass-fiber posts.

## Materials and methods

**Figure 1:**
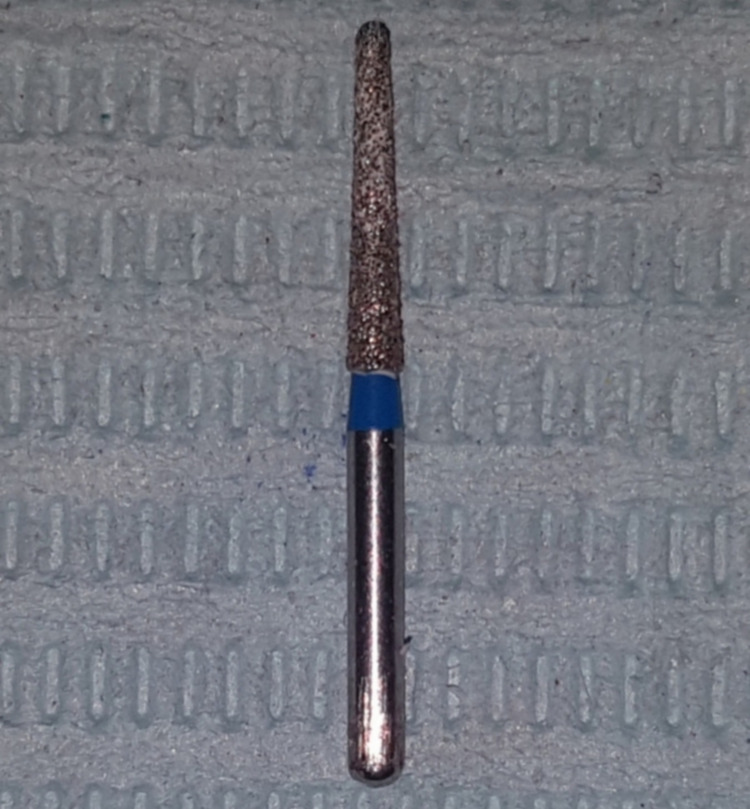
A diamond bur that was used as a diamond post

Forty human mandibular premolars indicated for orthodontic extraction were collected for this study. The inclusion criteria included teeth that were extracted from patients aged 18-30 years; had similar labiopalatal and mesiodistal dimensions (cementoenamel junction to apex distance: 14±1 mm, faciolingual dimension: 7±1 mm, mesiodistal dimension: 5±1 mm); had only one canal with complete root formation; did not have caries, severe curvature, cervical abrasion, fracture, or root resorption; and were not subjected to root canal treatment. The teeth were viewed under a magnification of 4x to determine whether all of the teeth matched the inclusion criteria. A digital caliper (Digimatic Calipers, Mitsutoyo, Japan) was used to measure the buccolingual, mesiodistal, and root lengths of all the chosen teeth. The teeth were cleaned with a hand scaler and then stored in 0.9% physiologic saline at room temperature (24-28°C) to prevent dehydration before and during experimental procedures. To mimic the clinical condition of a reduced tooth structure, the teeth were trimmed horizontally at 1 mm above the cementoenamel junction and perpendicular to their long axes using a low-speed diamond disc with water coolant, so that the lengths of the teeth after cutting are 15±1 mm. The working length was established at 1.0 mm short of the apical foramina. The step-down technique was used to prepare the root canals with (AF Gold, Fanta, China) using 5.25% sodium hypochlorite (NaOCl) as an irrigating solution. After completion of instrumentation, all specimens received a final flush of 17% ethylenediaminetetraacetic acid (EDTA) and dried with paper points. The root canals were obturated with gutta-percha and resin-based sealer (Adseal, META Biomed, South Korea) using the lateral condensation technique. Then, the root canal orifices of the roots were sealed with conventional glass ionomer (Harvard IonoGlas Fill, Harvard, Germany). Then, they were stored in distilled water at 37°C for 24 hours for the full setting of the sealer. Then, the teeth were distributed randomly into two groups of 20 teeth each. Twenty samples of endodontically treated teeth are restored with a diamond bur (DFS-Diamon, Germany) as a diamond post and a core, and 20 samples of endodontically treated teeth were restored with a glass-fiber post size 1 (NexPost, META Biomed, South Korea) and a core. The post-space preparation in the diamond post group was completed with Peeso Reamer (#1-2, Mani, Japan) to a depth of 10 mm under water coolant, leaving a 5±1 mm apical seal in the root canal. A root facer bur was used to form a space that fixed the diamond post to the coronal part of the canal. Then, a diamond bur was installed on a low-speed handpiece (NSK, Japan), and the canal was prepared at a speed of 1500 revolutions per minute under water coolant. The preparation of the canal to receive the post ends when the diamond bur moves easily in and out. Each bur used to prepare only three canals. After the preparation was done, the canal was washed using a 0.9% normal saline solution to eliminate the preparation residues. A diamond bur identical to the preparation bur was inserted into the canal and rotated manually 360 degrees with light pressure applied to prepare the walls of the endodontic canal to receive the diamond post, so that each bur is used to prepare only five canals. A diamond bur, identical to the preparation bur, was used as a diamond post (Figure 1).

**Figure 2:**
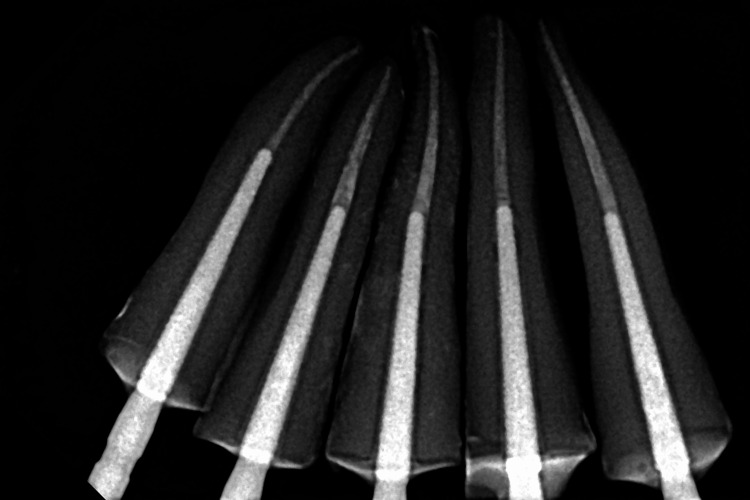
Periapical radiograph after cementation of diamond posts

**Figure 3:**
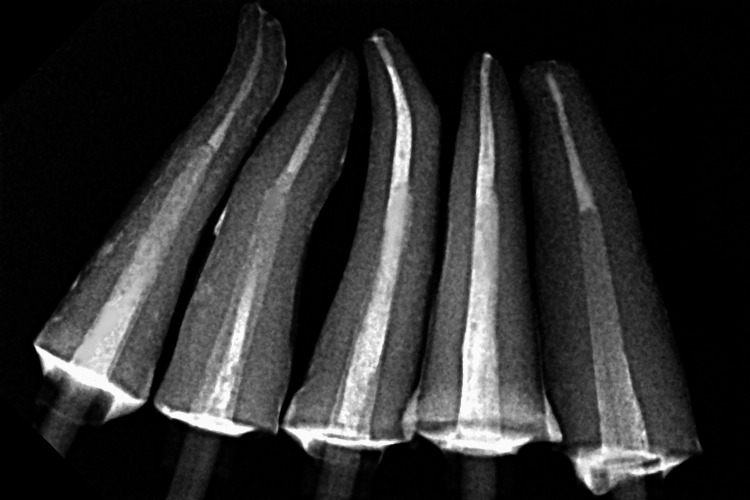
Periapical radiograph after cementation of glass-fiber posts

Using the kit's special low-speed drill, the post space in the glass-fiber post group was prepared to a depth of 10 mm under water coolant, leaving a 5±1 mm apical seal in the root canal. Post-space preparations were rinsed with 5.25% NaOCl. A final irrigation was carried out via distilled water, and post spaces were dried with paper points. For every sample, periapical radiographs were performed to verify the proper post-space preparation and to ensure the absence of any remnants of gutta-percha. Prior to luting steps, every post was marked at a distance of 10 mm from the apical end, equivalent to the length of post-space preparation. The full seating of the posts was properly confirmed in this manner. The canal was etched with 37% phosphoric acid (Eco-Etch, Ivoclar Vivadent, Liechtenstein) for 15 seconds and then rinsed with distilled water for 20 seconds. The canal was dried using paper points (Absorbent Paper Point, META Biomed, Korea). A dual-cure dentin bonding agent (Excite-FDSC, Ivovlar Vivadent, Liechtenstein) was placed on the root canal walls using an extra-fine brush. A paper point was used to eliminate any additional bonding material, followed by a gentle stream of air for five seconds, then light-cured for 20 seconds. Diamond posts were immersed in 70% alcohol for 60 seconds, then rinsed with distilled water, and dried with the air stream. A bonding agent (Tetric N-Bond, Ivovlar Vivadent, Liechtenstein) was applied to the surface of the diamond post with a brush and a gentle stream of air for five seconds and then light-cured for 20 seconds. Glass-fiber posts were cleaned with 70% alcohol, silane (Porcelain Primer, Bisco, USA) was applied and left to dry for a minute, and then a bonding agent (Tetric N-Bond, Ivovlar Vivadent, Liechtenstein) was applied to the surface of the post with a brush and a gentle stream of air for five seconds and then light-cured for 20 seconds. The cementation of the posts for both groups was done with dual-cure resin cement (NexCore, META Biomed, South Korea). By using a lentulo spiral, the cement was delivered to the root canal after being mixed in accordance with the instructions provided by the manufacturer. The post was then placed in its final location after being coated with a thin layer of cement. By applying finger pressure, each of the posts was placed into the prepared spaces to their full depth. A microbrush was used to remove the additional resin cement. Following its initial chemical polymerization, the cement was light-cured for 40 seconds using a light-curing unit (CuringPen-E, Eighteeth, China) with a 1200 mW/cm^2^ light intensity by positioning the light-curing unit's light-emitting tip on top of the post. The periapical radiographs of each specimen were taken to ensure complete seating of the posts without any gaps (Figures 2, 3).

**Figure 4:**
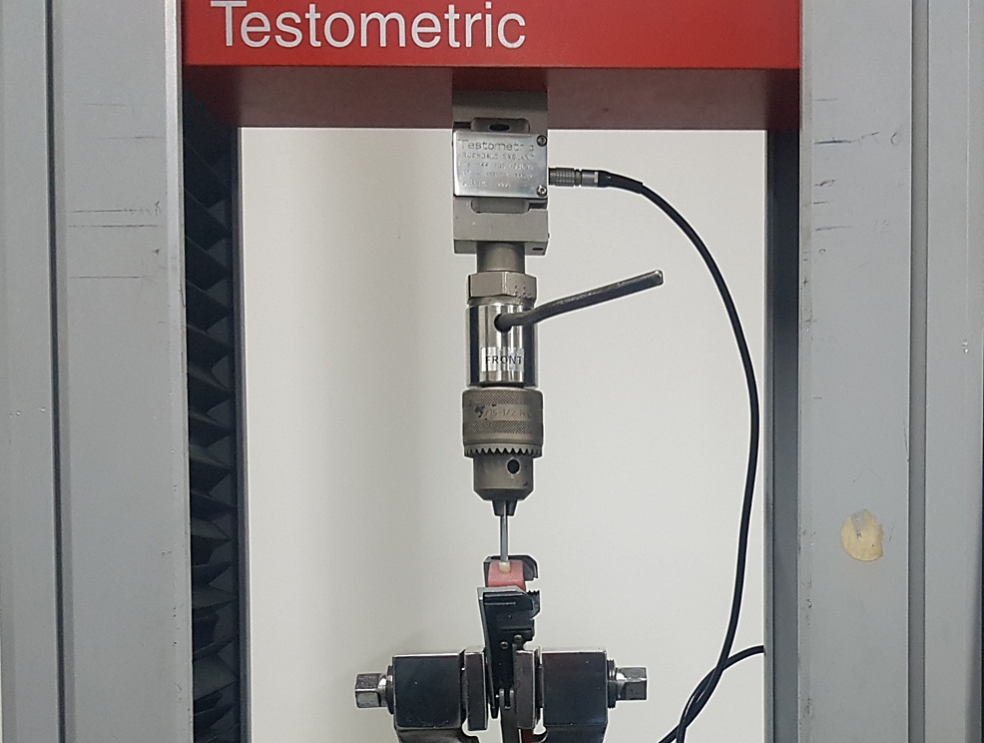
Application of force to the specimen in a universal testing machine

The core was built up with an etch-and-rinse bonding system (Tetric N-Bond, Ivovlar Vivadent, Liechtenstein) and a core build-up material (NexCore, META Biomed, South Korea). To guarantee uniformity in the size of the core of each specimen, prefabricated, transparent, light-transmissive celluloid crowns with an even height of 4 mm were utilized as molds. Self-curing acrylic was utilized to form acrylic blocks, which were inserted inside a transparent plastic mold (2 cm inner diameter, 2 cm height) that was modified to correspond with the mechanical testing device. Once each specimen was vertically inserted inside an acrylic block up to 2 mm beyond the cementoenamel junction, it was held under digital pressure until the material had begun to set. All specimens were kept in normal saline for 24 hours. The specimens were tilted at a 45° angle from the horizontal plane, and a compressive static load at a cross-head speed of 1 mm/min on the lingual incline of the buccal cusp at a distance of 2mm from the central fossa was applied using a universal testing machine (Testometric, M350-10KN, China) until visible or audible evidence of fracture was observed (Figure 4).

**Figure 5:**
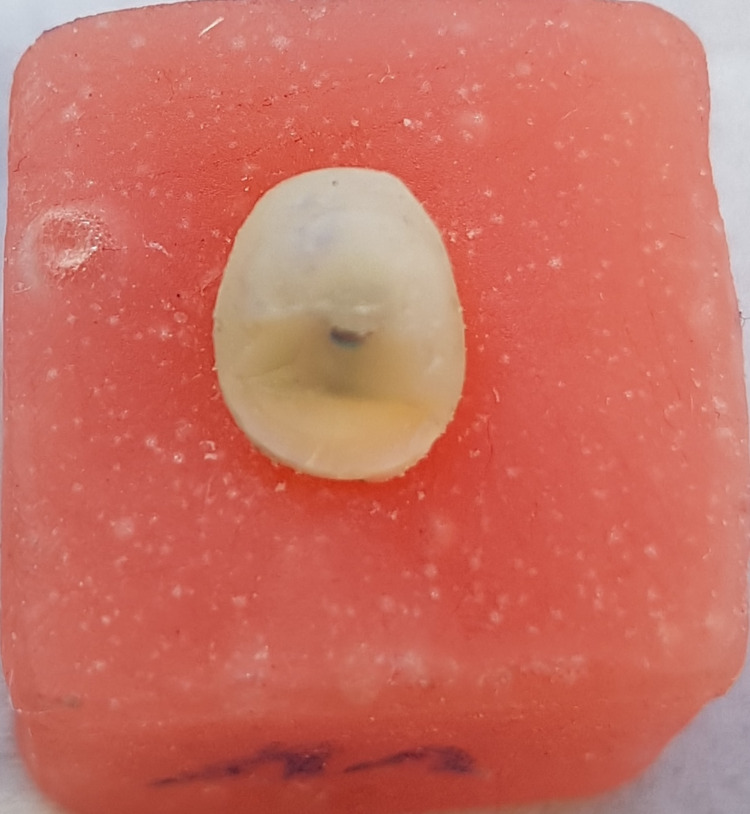
Favorable failure mode in the diamond post specimen

**Figure 6:**
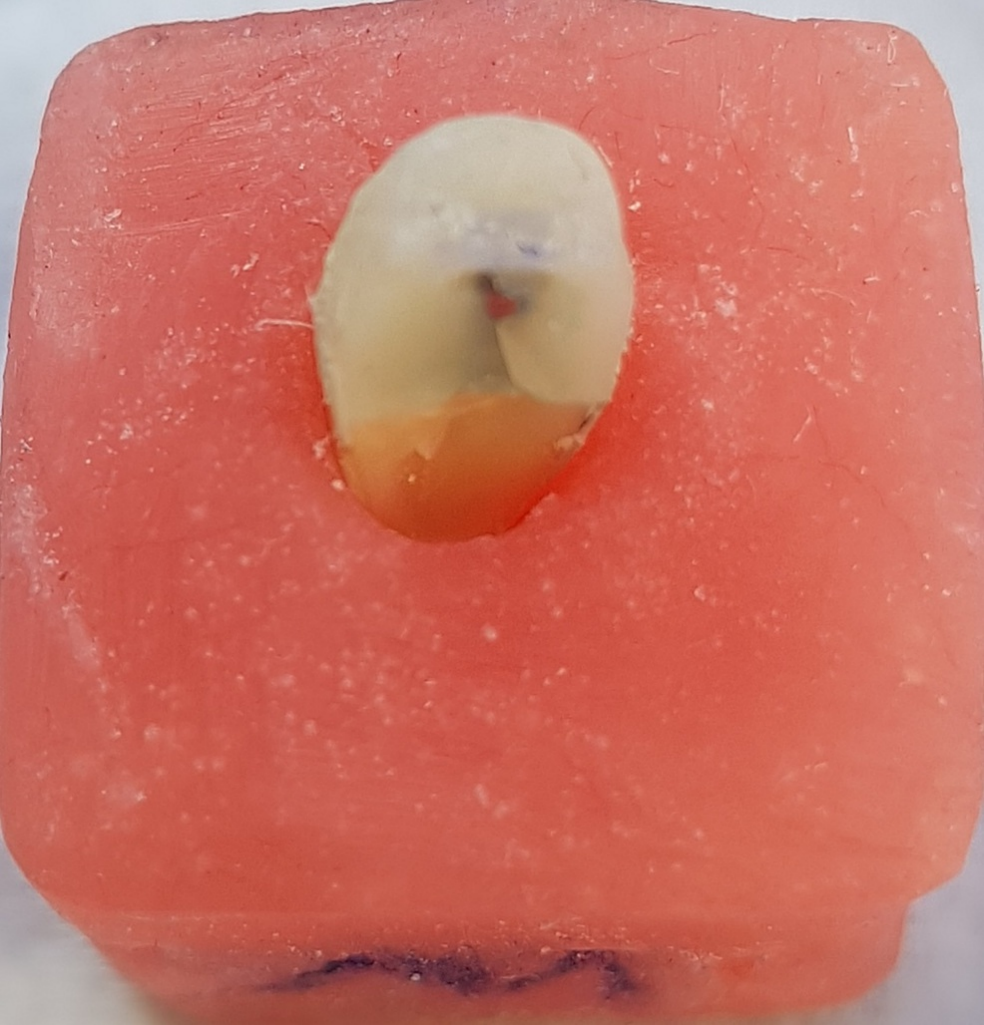
Unfavorable failure mode in the diamond post specimen

**Figure 7:**
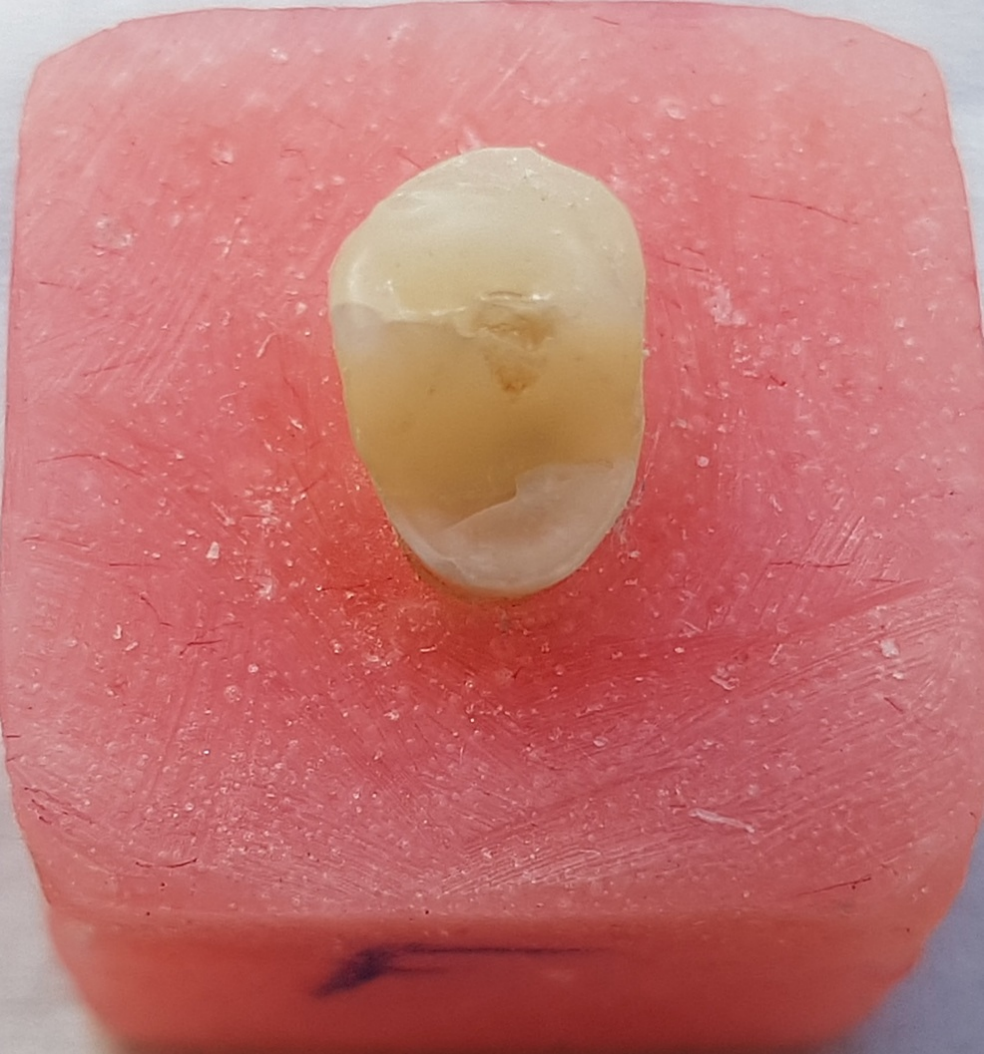
Favorable failure mode in the glass-fiber post specimen

**Figure 8:**
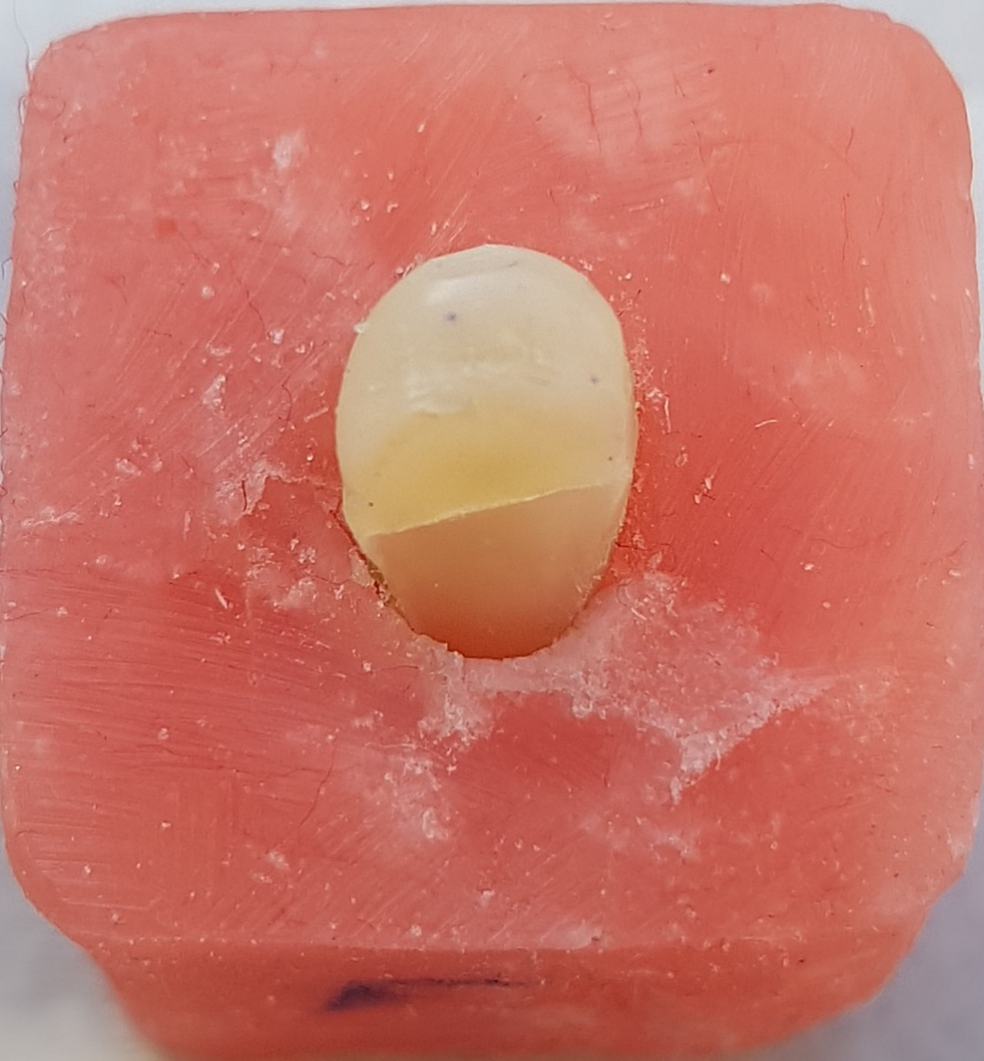
Unfavorable failure mode in the glass-fiber post specimen

The fracture strength was measured in Newton, and the mode of failure was recorded as the fracture above the acrylic block level was considered favorable and the fracture below the acrylic block level was considered unfavorable ​(Figures 5-8). Two impartial observers observed it by eye inspection.

The normal distribution of the fracture resistance values was confirmed by using the Kolmogorov-Smirnov test. The statistical analysis of fracture resistance between the two groups was performed by t-test. For the mode of failure, the statistical analysis was performed by the chi-square test. For all tests, the significance level was determined at 0.05. SPSS for Windows, version 16.0 (released 2007, SPSS Inc., Chicago) was used to perform the statistical analysis.

## Results

In all the tested specimens, the diamond post group exhibited higher fracture resistance than the glass-fiber post group. A t-test was used for a comparative evaluation of the mean fracture resistance. The test showed that when comparing the diamond post group with the glass-fiber post group, it was found to be statistically significant (Table 1).

**Table 1:** Comparison of fracture resistance between the diamond post group and glass-fiber post group * p-value < 0.05 statistically significant

Posts type	Quantity	Mean fracture resistance (in Newton)	Standard deviation	Minimum value	Highest value	t-test value	P value	Significant/non-significant
Diamond post	20	435.80	80.25	305	572.7	5.979	0.000*	Significant
Glass-fiber post	20	300.60	61.53	208.2	415			

The mode of failure in the diamond post group was as follows: 55% of the specimens had favorable failure modes, and 45% of the specimens had unfavorable failure modes. In the glass-fiber post group, 75% of the specimens had favorable failure modes, and 25% of the specimens had unfavorable failure modes. The comparison of the mode of failure was performed among both groups with the chi-square test, and the results showed that there was no statistically significant difference in terms of the failure mode between the two groups (Table 2).

**Table 2:** Comparison of the mode of failure between both groups * p-value > 0.05 statistically insignificant

Group	Frequency			Percentage		Chi-square test		
	Favorable	Unfavorable	Total	Favorable	Unfavorable	Chi value X^2^	P value	Significant/non-significant
Diamond post	11	9	20	55.0%	45.0%	1.758	0.185*	Non-significant
Glass-fiber post	15	5	20	75.0%	25.0%			
Total sample	26	14	40	65.0%	35.0%			

## Discussion

Tooth fracture is one of the most common reasons for tooth loss [10]. According to certain research, teeth that have undergone endodontic treatments are more susceptible to fractures [1,11]. As stated by Ellis et al., significant endodontic damage to the tooth structure during the instrumentation and a drop in the quantity of wetness make teeth weaker and more brittle [11]. The amount of preserved dentin is found to be a determining factor in the ability of a tooth restored with a post to resist fractures. The fracture resistance is increased when more dentin is left [12]. The dentist's primary objective is to maintain the intact tooth tissue, and a conventional approach is to be taken during the access cavity and instrumentation and be as conservative as possible to save the residual dental structure. Glass fibers that are directed just one way and embedded in a resin matrix constitute glass-fiber posts, which reinforce them while not modifying their flexibility. As glass fibers disperse stress throughout a large surface area, the load limit at which the post starts to exhibit signs of fractures is raised [9]. There are many types of prefabricated metal posts, such as stainless steel and titanium, that are used for restoring endodontically treated teeth. In this study, a new technique was used that aims to use a diamond bur as a prefabricated metal post to restore endodontically treated teeth. It possesses several characteristics, including the roughness of the diamond part, which improves its engagement with dentin. The fine voids on the diamond part of the bur allow the cement to be embedded inside it. It is unthreaded, and it has great diversity in its shapes and dimensions. It provides ease of use, and there is no need for additional tools for preparation. The possibility of adjusting the coronal part of the bur stabilizes the restoration within the clinic. It has a low cost compared to other types of posts. However, it has a higher modulus of elasticity compared to dentin and does not match the tooth color. It is intended that the optimal post system will be less susceptible to fracture compared to the normal biting loads, but it must not be so rigid that it fractures the root irreparably, so the fracture resistance of the tooth restored with a post is considered an important factor to be assessed [13]. The goal of the current *in vitro* research was to assess the fracture resistance and mode of failure of endodontically treated teeth restored with diamond posts and glass-fiber posts. Since artificial teeth cannot replicate natural dentin or lessen levels of loads that can cause fracture, natural teeth were utilized in this study to mimic clinical situations [14]. Mandibular premolars were used in this study. According to a previous study, higher bite forces are applied to posterior teeth as opposed to anterior teeth [3]. Moreover, premolars have a greater probability of being exposed to lateral forces compared to molars. Moreover, there is usually just one root on the mandibular premolars [3]. Mandibular premolars have also been used in a number of research studies to evaluate the fracture resistance of the post system and have been considered suitable for this purpose [5,15,16]. Since the dimension of the teeth was shown to be a crucial factor in the specimen's ability to withstand fracture, it was meticulously chosen for this research to be uniform [17]. To replicate the clinical circumstance of a severely decayed tooth and demonstrate the clinical impact of the post and core ability to withstand fracture, only 1 mm of coronal structure for every specimen was kept [18]. Unlike Gates Glidden burs, nickel-titanium (Ni-Ti) files had no effect on the teeth's ability to withstand fracture when the coronal third of the canal was preflared [19]. Therefore, in this study, the canals were prepared using the step-down technique with heat-treated Ni-Ti files. A lateral condensation technique with gutta-percha and resin sealer was utilized because it was found that this technique and the Thermafil technique had the best fracture resistance between the different root canal filling methods [20]. Many materials used for direct core build-up include amalgam, composite, and glass ionomer cement [10]. Amalgam has good mechanical characteristics, but it does not match the tooth color, and postponing crown preparation is necessary to give the material enough time to set [3]. The composite material matches the tooth color and can be prepared at the same visit [3]. Drawbacks include polymerization shrinkage and microleakage [21]. Glass ionomers are not strong enough to be used as a core build-up material [21]. The use of core materials for cementing posts is an effective method [22]. Also, less technique sensitivity appears, fewer steps are needed, and there is no longer a chance that the cement and core material cannot be used with each other [23]. The elastic modulus of the core build-up material (NexCore, META Biomed, South Korea) is 14.2 GPa, according to the manufacturers, which is greatly equivalent to the elastic modulus of dentine (15 GPa), and it achieved a monoblock between the tooth, fiber post, and core that might evenly disperse biting loads and lessen stress as a result [24]. Hence, the core build-up material was used to cement the post and build up the core. In order to uniformize the dimension of the core in each specimen, a cellulose template was utilized. According to prior studies, a crown is not placed over the specimen, as it could hide the impact of the post and core systems [25,26]. In addition, consistent with earlier research, the samples were mounted rigidly without periodontal copying, which could avoid any movement of the teeth throughout testing [25,27]. In the current study, specimens were placed immediately into the acrylic mold, allowing the dental structure to mainly receive the load. According to a previous study, when the samples were mounted without periodontal simulation, this may have led to a decreased failure load compared to what might have been noticed clinically [27]. However, another study found no difference in the fracture resistance between teeth mounted rigidly or with periodontal simulation under a static loading test [28]. In accordance with a prior study, samples were placed 45 degrees off the horizontal plane and then subjected to a constant load [29]. Meanwhile, various angles from the horizontal plane were utilized in previous research, such as a 30° angle [16], a 60° angle [15], and a 90° angle [5]. The current study utilized a cross-head speed of 1 millimeter per minute. In the published research, there was an extensive variation of cross-head speeds from 0.5 mm/min [4,5,15] to 1 mm/min [2,16] and 5 mm/min [8]. According to the study's findings, neither the fracture resistance nor the mode of failure of either post system responds in the same way when subjected to identical test conditions.

The diamond post group exhibited higher fracture resistance than the glass-fiber post group. A comparative evaluation of the mean fracture resistance between the diamond post group and the glass-fiber post group was found to be statistically significant. The reason for the superiority of the diamond post can be attributed to the roughness of the diamond part, which improves its engagement with dentin. Moreover, fiber posts bow at less force, permitting loads to reach the tooth more quickly and increasing the likelihood that the tooth may fail under minimal compressive levels [25]. In the present research, the mode of failure was assessed. The specimen was inserted 2 mm beneath the cementoenamel junction in the acrylic mold, imitating the level of alveolar bone [14]. According to whether the specimen's fracture occurred above or underneath the level of the inserted acrylic, the mode of failure was classified as either favorable or unfavorable. The ability to access and sufficient quantity of residual tooth tissue present in fractures above the inserted acrylic level were deemed favorable, as they were able to be restored. Because the procedure of restoration would be challenging, the specimen fracture beneath the acrylic level was deemed unfavorable [8]. According to the comparison between the diamond post group and the glass-fiber post group, although the difference was not significant, the glass-fiber post group exhibited a more favorable failure mode. The reason for the occurrence of more favorable failure modes in the glass-fiber post group may be that once the post and core system are subjected to load, an extremely stiff post will be unable to continue the distortion due to flexibility but instead generate a concentrated compression peak across the root, which ultimately results in the root fracture. Conversely, the dentin's modulus of elasticity and the glass-fiber post are comparable, so the glass-fiber post disperses additional loads through the restoration while little stress is applied to the dentin, showing favorable fractures [4]. Furthermore, the monoblock form of restoration created by the glass-fiber post, luting cement, and the core efficiently transfers and disperses loads throughout the structure of the tooth [2]. The current *in vitro* research provides details regarding the biomechanical behavior of two types of posts that are potentially helpful in post choice and improving the longevity of root canal-treated teeth *in vivo*. Such a kind of *in vitro* study obviously does not take into account all of the clinical circumstances. It was nonetheless based on studies utilizing post and core that had already been documented throughout the academic publications.

The limitations of this study were that it was performed *in vitro* by using static loading at a single point; this may not accurately reflect the actual *in vivo* condition, as the masticatory forces are multidirectional and frequently applied to a wider area. Also, to mimic the intraoral situation, further *in vitro* research ought to take the aging process into account, as thermocycling could have an impact on the outcomes. In addition, only mandibular premolars were used; therefore, these findings could only be limited to this group of teeth.

## Conclusions

Within the limitations of this study, it could be concluded that diamond posts showed higher fracture resistance than glass-fiber posts. Glass-fiber posts showed more favorable failure modes but were statistically insignificant compared to diamond posts. Further studies are required to assert the outcomes of this study and before anything can be considered definitive for clinical use.
